# The long-quarantined case of COVID-19 with prolonged viral shedding and intermittent fever for more than 70 days

**DOI:** 10.2217/fvl-2020-0423

**Published:** 2021-02-09

**Authors:** Eijiro Yamada, Emi Ishida, Yasuyo Nakajima, Kazuhiko Horiguchi, Shunichi Matsumoto, Shuichi Okada, Wataru Kamitani, Masanobu Yamada

**Affiliations:** 1 ^1^Department of Medicine & Molecular Science, Gunma University Graduate School of Medicine 3-39-15 Showa-machi, Maebashi, Gunma 371-8511, Japan; 2 ^2^Department of Infectious Diseases & Host Defense 3-39-15 Showa-machi, Maebashi, Gunma 371-8511, Japan

**Keywords:** COVID-19, favipiravir, infectivity, interstitial pneumonia, reverse transcriptase-PCR, viral shedding, WHO standards

## Abstract

A 79-year old Japanese woman was diagnosed with coronavirus disease (COVID-19), caused by SARS coronavirus 2 (SARS-CoV-2), based on a positive reverse transcription-PCR (RT-PCR) test result. Chest computed tomography revealed mild interstitial pneumonia. She had intermittent persistent inflammatory reactions with fever. Laboratory findings and RT-PCR test results showed SARS-CoV-2 positivity for more than 70 days. To the best of our knowledge, this relatively mild case has the longest duration of viral shedding recorded, as confirmed by RT-PCR analysis. This case demonstrates that the viral shedding in COVID-19 can be prolonged, even in mild disease, and highlights the difficulties in distinguishing viral shedding from SARS-CoV-2 infectivity.

The outbreak of SARS coronavirus 2 (SARS-CoV-2) has resulted in a global pandemic, affecting all countries worldwide, including Japan [1]. According to retrospective analyses of clinical and epidemiological characteristics of patients with coronavirus disease (COVID-19), viral shedding is considered to last for an average of 12–20 days [2]. While prolonged viral RNA shedding has been reported to correlate with the severity of COVID-19 [3], the longest duration reported to date for mild cases with no significant past history and no invasive mechanical ventilation is 60 days [4].

## Case report

**Figure 1.**
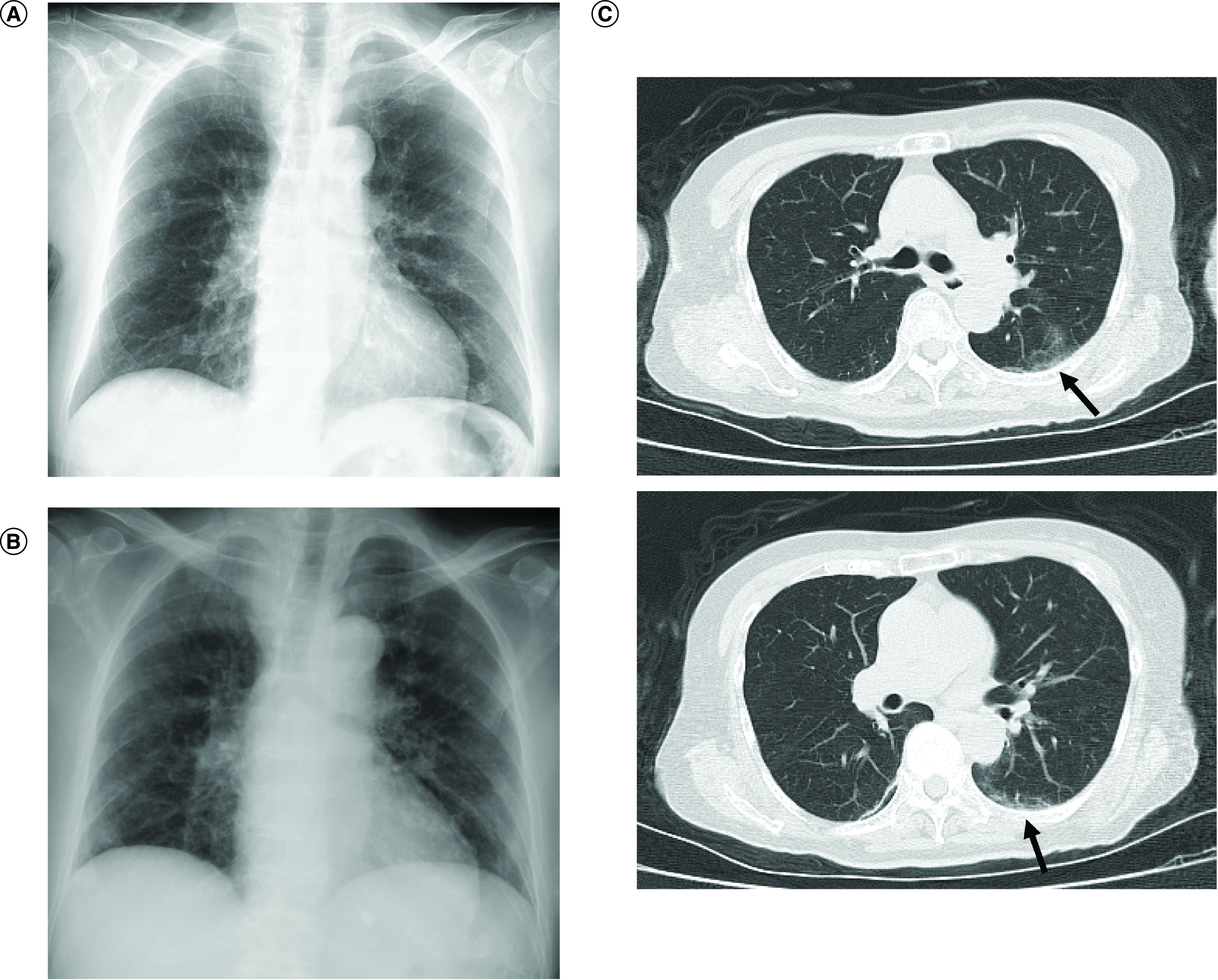
Chest x-ray and computed tomography. **(A)** Plain chest radiograph at the erect position on day 1. **(B)** Chest radiograph in the supine position on day 27. **(C)** Chest computed tomography on day 1. The black arrows indicate ground-glass opacities, suggesting interstitial pneumonia.

**Figure 2.**
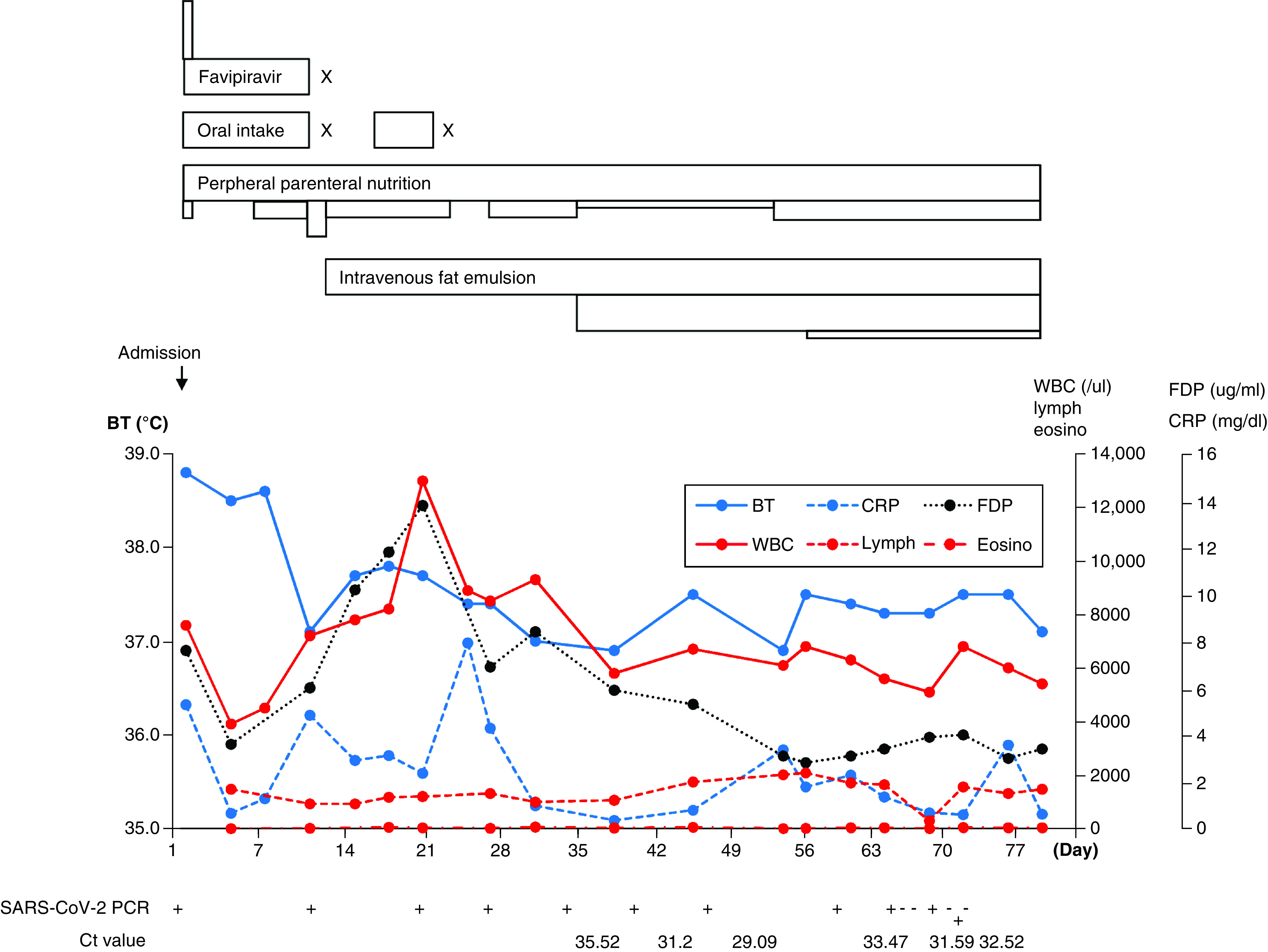
**Progression of treatment and laboratory data.** Top image: each rectangle indicates the treatment performed. The length indicates the period of each treatment, and the height indicates the dose. The scale is shown in the graph below. X indicates the day we stopped the treatment. The changes in each laboratory findings are shown in the line graph. The day we performed the RT-PCR test is indicated by a plus sign below the graph. All RT-PCR test results were positive during the period shown in the figure. BT: Body temperature; CRP: C-reactive protein; Eosino: Eosinophils; FDP: Fibrin degradation product; Lymph: Lymphocyte; RT-PCR: Reverse transcription-PCR; WBC: White blood cell.

**Figure 3.**
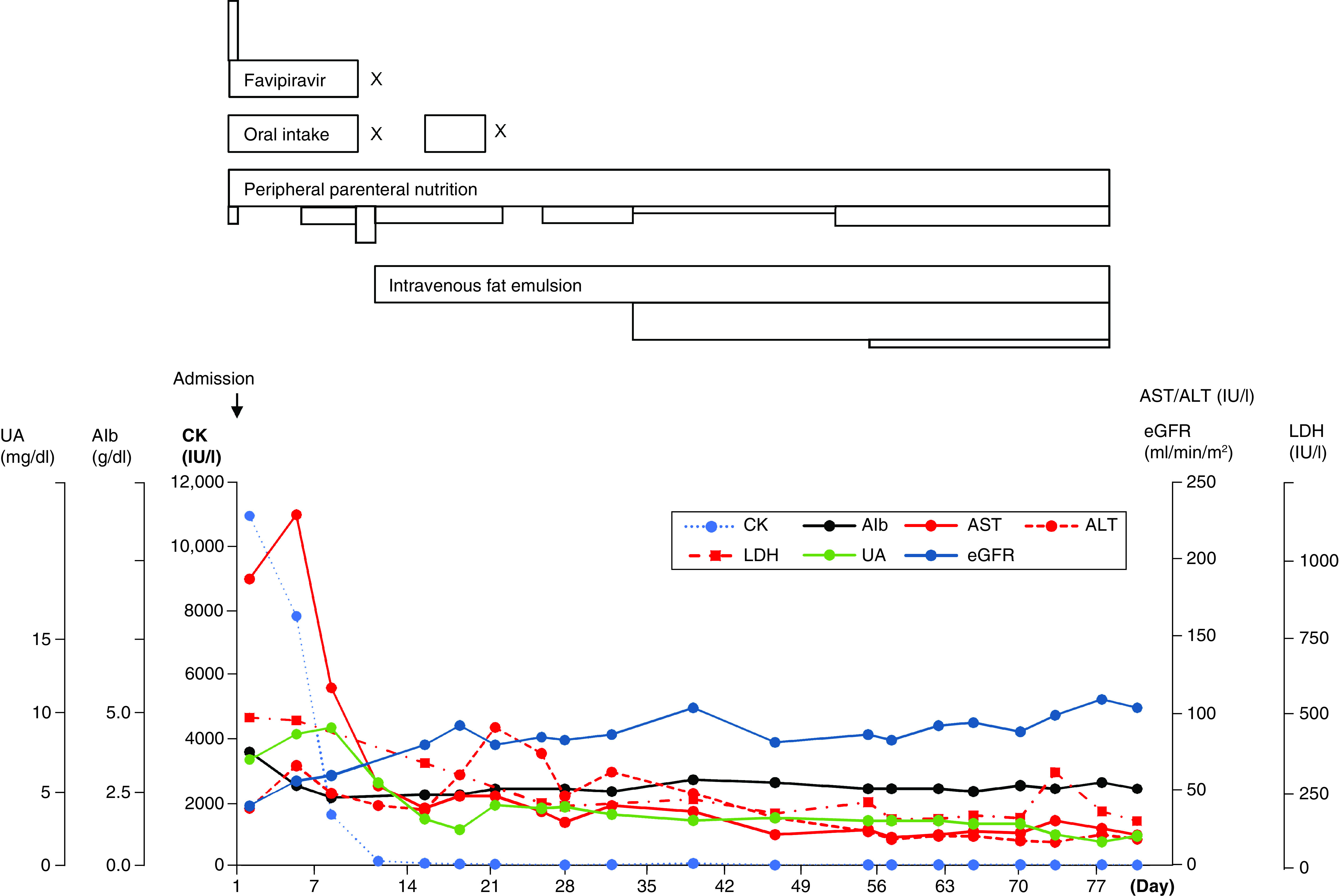
Progression of treatment and laboratory data (continued from Figure 2). Top image: same as Figure 2. The changes in other laboratory findings are shown below. Alb: Albumin; ALT: Alanine aminotransferase; AST: Aspartate transaminase; CK: Creatine kinase; eGFR: Estimated glomerular filtration rate; LDH: Lactate dehydrogenase; UA: Uric acid.

A 79-year old woman presented with a high fever and mild cough. Since she lived in a nursing home where some of the residents were diagnosed with COVID-19, she was immediately transferred to our hospital without performing a reverse transcription-PCR (RT-PCR) test for SARS-CoV-2. All the residents and employees of the nursing home were admitted to hospital. In total, 68 individuals who lived or worked in the nursing home were diagnosed with COVID-19, indicating a cluster outbreak of the disease. The first patient, in whom the COVID-19 diagnosis was confirmed by RT-PCR 1 day before our patient was admitted, had a high fever for the previous 4 days. On our patients admission, RT-PCR using a nasal swab sample confirmed the presence of SARS-CoV-2; hence, a diagnosis of COVID-19 was made. Of note, the patient had a high fever of approximately 39.0°C and pulmonary sounds. She was a nonsmoker and had been taking an antidepressant since she was 65 years old. Moreover, her BMI was 16.0 kg/m^2^, and she had a history of fractured neck of the femur twice at ages of 75 and 76 years, demonstrating frailty. On admission, laboratory test results showed an increased C-reactive protein level (50.3 nmol/l), without an increase in the white blood cell count. Generally, it has been observed that lymphocyte counts decrease in COVID-19 patients [2,5]. Interestingly, her creatine phosphokinase level markedly increased to more than 160 μkat/l, which is consistent with COVID-19 [5]. Arterial blood gas analysis revealed that her oxygenation capacity was well maintained on room air. While a chest radiograph revealed a few inflammatory changes, computed tomography showed mild interstitial pneumonia (Figure 1). Her clinical course is shown in (Figures 2 & 3). On diagnosis, she was administered favipiravir, which is used for the treatment of COVID-19 in Japan. However, we were forced to discontinue the administration of all oral drugs, including favipiravir, due to dysphagia on day 10. Although, her fever and laboratory results improved after treatment with favipiravir, she aspirated several times and had intermittent fever, indicating possible aspiration pneumonia. Therefore, she underwent fasting management and was administered peripheral parenteral nutrition. After favipiravir treatment, RT-PCR tests were occasionally conducted to examine for viral shedding, but it showed positive results for more than 60 days after diagnosis. In Japan, the guidelines for the discharge of symptomatic patients with COVID-19 have been frequently revised according to WHO standards [6]. The guidelines allowed the discharge of patients 10 days after symptom onset and 72 h after symptoms resolution. Since she had intermittent fever, she could not be discharged until two RT-PCR tests using nasal swab samples confirmed the absence of viral shedding. Finally, RT-PCR performed 73 days after her first positive test revealed negative results.

## Discussion

Prolonged SARS-CoV-2 RNA shedding is not a rare phenomenon. The longest duration of viral shedding reported for mild cases without invasive mechanical ventilation is 60 days from the first positive RT-PCR result [4]. Additionally, we found another case report that described more than 70 days of viral shedding from symptoms onset, but more than 60 days after obtaining positive PCR results [7]. Here, we have reported a case of confirmed persistent viral shedding of SARS-CoV-2 based on RT-PCR testing for more than 70 days, which, to the best of our knowledge, is the longest duration reported to date for mild cases.

The following factors are associated with prolonged viral shedding in patients with COVID-19: male sex; delayed hospital admission after illness onset and; invasive mechanical ventilation [8]. Surprisingly, none of these factors were applicable in our case, which pertained to a woman who was admitted on the day of fever onset and did not require invasive mechanical ventilation. Moreover, while prolonged viral shedding has been observed in severe cases, our case was a mild case with only a high fever and no respiratory symptoms, despite abnormal laboratory and chest computed tomography findings [8]. Additionally, despite being frail, she had no complications such as respiratory diseases or diabetes mellitus, which are now considered risk factors for severe COVID-19 [5,6]. This indicates that prolonged viral shedding in patients with COVID-19 is unpredictable and may even occur in mild cases. Old age is a significant factor associated with prolonged viral shedding [9]. Although, prolonged viral shedding has been observed in many cases, limited information is available on prolonged viral shedding in immunocompromised patients. It has recently been reported that prolonged viral shedding can be observed after immunosuppressive therapy for cancer [10]. Moreover, it was noted that a diminished CD8^+^ T-cell response, which is seen in people with frailty [11], correlates with prolonged viral shedding [12]. Therefore, frailty might explain the prolonged viral shedding in our case.

The duration of viral shedding is often considered to determine the appropriate period of isolation as it could be a marker of infectivity [13]. Considering this, the Japanese guidelines previously mandated two negative RT-PCR tests using nasal swabs samples 24 h apart in accordance with the WHO standards. However, a positive RT-PCR result does not necessarily indicate the potential for viral transmission because the amount of SARS-CoV-2 viral RNA detected does not always indicate greater infectivity. Therefore, the conditions for discharge were revised as follows: 10 days after the symptom onset and 72 h after the symptom resolution. Since our patient had frequent aspirations and fever, indicating possible aspiration pneumonia, it was difficult to distinguish pneumonia from COVID-19 and aspiration. Therefore, although the Japanese guidelines for quarantine were revised, two negative RT-PCR test results using nasal swab samples were required to confirm the absence of viral shedding. There is limited understanding of the significance of viral shedding of SARS-CoV-2 with respect to its infectiousness and reactivation. This has resulted in a clinical dilemma. It is crucial to have a clearer understanding of these fundamental concepts considering the multiple waves of COVID-19 worldwide, including in Japan [1]. Detecting seroconversion onset could be a clue to overcoming these problems. Recently, it has been reported that viral shedding of SARS-CoV-2 drops rapidly to undetectable levels on seroconversion and a serum neutralizing antibody titer is independently associated with noninfectious SARS-CoV-2 [14]. Our patient had both increased IgG and IgM levels, indicating seroconversion. Further studies are needed to confirm this hypothesis.

On diagnosis, our patient was administered favipiravir, which is regularly used for the treatment of COVID-19 in Japan. However, we were forced to discontinue the medication due to dysphagia on day 10. Favipiravir is a new type of RNA-dependent RNA polymerase inhibitor that has been approved for influenza treatment [15]. Theoretically, it has potential antiviral action against the RNA virus SARS-CoV-2. Although, clinical trials of favipiravir for COVID-19 treatment are ongoing, its efficacy is not yet confirmed. However, some reports have demonstrated its effectiveness, particularly in increasing the cumulative virus elimination rate. However, in our case, no obvious effect on virus elimination was observed; therefore, more studies are needed to understand COVID-19 treatment with favipiravir and other potential drugs.

In summary, we have presented a case of persistent viral shedding of SARS-CoV-2 based on RT-PCR testing for more than 70 days. To the best of our knowledge, this is the longest duration of viral shedding reported worldwide thus far. More importantly, our case was a mild case with no complications. The patient was quarantined for more than 70 days since she had fever that could not be distinguished from aspiration pneumonia. It is critical to understand the mechanisms behind persistent viral RNA shedding and the possibility of viral reactivation to help manage COVID-19 effectively.

## Future perspective

This case demonstrated that the viral shedding in coronavirus disease can be prolonged and that there are difficulties in distinguishing viral shedding from SARS-CoV-2 infectivity. While there is a persistent lack of understanding of the significance of shedding of SARS-CoV-2 with respect to its infectiousness and reactivation, clinical problems will ensue.

**Summary points**

**Background**

- Coronavirus disease (COVID-19), caused by SARS-coronavirus-2 (SARS-CoV-2), has become a public health emergency in all countries worldwide, including in Japan.
- Viral shedding, which is often considered to determine the period of isolation as it could be a marker of infectivity, is considered to last for an average of 12–20 days; the longest duration of viral shedding for mild cases reported to date is 60 days.

**Case summary**

- A 79-year old Japanese woman was diagnosed with COVID-19 using reverse transcription-PCR (RT-PCR) tests. Chest computed tomography revealed mild interstitial pneumonia. She had persistent mild inflammatory reactions with fever, but she did not require invasive mechanical ventilation. Laboratory findings and RT-PCR test results showed SARS-CoV-2 positivity more than 70 days.

**Conclusion**

- To the best of our knowledge, this case reports the longest duration of viral shedding in mild cases. This case demonstrates that viral shedding in COVID-19 can be prolonged, even in mild cases and highlights the difficulties in distinguishing viral shedding from SARS-CoV-2 infectivity.
